# Impact of Enzyme–Microbe Combined Fermentation on the Safety and Quality of Soy Paste Fermented with Grass Carp By-Products

**DOI:** 10.3390/foods14010106

**Published:** 2025-01-02

**Authors:** Jing Yang, Zihan Li, Xinping Lin, Sufang Zhang, Chaofan Ji

**Affiliations:** SKL of Marine Food Processing & Safety Control, National Engineering Research Center of Seafood, Collaborative Innovation Center of Seafood Deep Processing, School of Food Science and Technology, Dalian Polytechnic University, Dalian 116034, China; 221720860001039@xy.dlpu.edu.cn (J.Y.); 231720951351095@xy.dlpu.edu.cn (Z.L.); linxp@dlpu.edu.cn (X.L.); zhangsf@dlpu.edu.cn (S.Z.)

**Keywords:** fish by-products, starter cultures, food fermentation, condiments

## Abstract

Freshwater fish processing produces 30–70% nutrient-rich by-products, often discarded or undervalued. Grass carp by-products, rich in protein, offer potential as raw materials for fermented seasonings. This study explores the use of these by-products—specifically, minced fish and fish skin—in soybean fermentation to evaluate their effects on the quality of the resulting seasonings. *Tetragenococcus halophilus* was used as a starter culture alongside food-grade protease to assess their combined impact on the safety and flavor of soy fish paste and soy fish skin paste. The findings revealed that natural fermentation resulted in higher protein hydrolysis in soy fish skin paste compared to soy fish paste. Across all fermentation conditions, amino acid nitrogen levels increased, while total volatile basic nitrogen levels decreased in both pastes, indicating improved quality. Additionally, microbial fermentation significantly reduced biogenic amine content in soy fish paste, enhancing safety. Enzymatic fermentation further enriched the flavor of both pastes by boosting key compounds such as 2-methylbutanal and ethyl acetate. Notably, enzyme-microbe co-fermentation harnessed the strengths of both methods, achieving improved safety and enhanced flavor profiles while elevating overall product quality. These findings suggest a promising way to transform freshwater fish by-products into high-value condiments, advancing sustainable food processing.

## 1. Introduction

Fish by-products, comprising bones, skin, and other non-filet parts, make up to 70% of fish biomass [1]. These by-products are abundant in proteins, lipids, polysaccharides, and other valuable nutrients. However, their primary use remains in low-value products like animal feed, and many are discarded. This practice results in both resource waste and increased environmental pressure. Their utilization will support sustainability by reducing waste, maximizing resource efficiency, and contributing to the food industry. In recent years, fermentation has been regarded as a processing method that promotes the release of nutrients from fish by-products, enhancing their value [2].

Soy paste is a traditional fermented condiment made primarily from soybeans and salt, which is widely used in Asian countries. The fermentation process involves two stages: in the first stage, *Aspergillus oryzae* is used to ferment the steamed soybeans; in the second stage, a brine solution is added, allowing a naturally formed microbial community to further ferment the paste. Soy sauce is the liquid seasoning extracted from the former. In recent years, soybean prices have been steadily increasing [3]. If part of the raw materials in soy paste fermentation can be substituted without compromising quality, it would reduce costs while maintaining a stable product quality.

Grass carp (*Ctenopharyngodon idella*) is one of the most important freshwater fish species consumed in China and other Asian countries [4]. Crass carp can be used for producing surimi [5], leaving a large quantity of fish mince and fish skin. Due to their high protein content and irregular shapes, these by-products are ideal raw materials for fermented seasoning production. Research has already been conducted on fermenting low-value fish mixed with soybeans to enhance the umami of seasoning sauces [6]. However, for fish by-products, the chemical composition varies significantly across different parts, and this variation should be considered when using them as raw materials for fermented sauces. In addition, current fermentation methods for fish-based seasonings often use the fish’s own autolytic enzymes or spontaneous fermentation by microbial communities to break down proteins into peptides and amino acids [6,7]. To improve the commercial use of freshwater fish by-products, several challenges need to be overcome. A key focus is the development of novel microbial strains, enzymes and related process method for fermentation and hydrolysis, with the goal of controlling the fermentation process, enhancing flavor compounds, and minimizing harmful substances [8].

This study aims to explore the use of grass carp by-products, specifically mince and skin, as alternative raw materials to soybean for the production of soy fish paste and soy fish skin paste. This research focuses on evaluating the feasibility of these replacements while identifying suitable starter cultures and proteases to optimize the safety and quality of the final products. Key safety indicators include biogenic amines and volatile basic nitrogen, while quality assessment also considers lipid oxidation indices, sensory analysis, and the composition of volatile compounds in the paste. These findings aim to support the development of fermentation and hydrolysis techniques tailored to specific raw materials, methods for flavor enhancement, and quality control standards, enabling the sustainable production of eco-friendly seasonings from freshwater fish by-products.

## 2. Materials and Methods

### 2.1. Raw Materials and Chemical Reagents

Soybeans were purchased from Zhennong Grain Planting Company (Yilan, Heilongjiang, China). Wheat was purchased from Changlong Trading Company (Zhoukou, Henan, China). Soy sauce koji (*Aspergillus oryzae*) was obtained from Weihengmei Trading Company (Suqian, Jiangsu, China). Table salt was purchased from Zhongyan Company, Shanghai, China. Grass carp was sourced from a local market in Dalian, China. Grass carp skin was obtained from Yu Zhige Food Materials Company in Guangdong, China.

Formaldehyde, sodium hydroxide, sodium bicarbonate, boric acid and sodium chloride were purchased from Damao Chemical Reagent Company, Tianjin, China. Hydrochloric acid, 25% ammonia solution, methanol, anhydrous potassium carbonate, and gum arabic were obtained from Aladdin Biochemical Technology Company, Shanghai, China. Biogenic amine standards (spermine, spermidine, tyramine, histamine, cadaverine, putrescine, phenethylamine, and tryptamine), thiobarbituric acid, dansyl chloride, bromocresol green–methyl red indicator, and pepsin were obtained from Macklin Biochemical Technology Company, Shanghai, China. Acetonitrile was purchased from Sigma-Aldrich Company, Shanghai, China. All the above chemical reagents are of analytical or chromatographic grade. Papain and neutral protease were purchased from Yuan Ye Bio-Technology Company, Shanghai, China. *Tetragenococcus halophilus* was isolated from naturally fermented soy sauce and is available from the China Center of Industrial Culture Collection (CICC 10286). MRS Broth was purchased from Qingdao Hi Tech Industrial Park Haibo Biotechnology Co., Ltd., Qingdao, China.

### 2.2. Preparation of Soy Fish Paste and Soy Fish Skin Paste

Firstly, koji was prepared by inoculating cooked soybeans with the fermentation culture *Aspergillus oryzae*. Wheat was washed, roasted, and ground into powder, while soybeans were soaked for 8–9 h, steamed at 121 °C for 15 min, and then cooled. The wheat powder, soybeans, and *A. oryzae* powder (0.2%, *w*/*w*) were combined and fermented at 30 °C for 68 h.

For soy fish paste, grass carp minced meat is mixed with prepared koji (koji:minced fish meat = 2:1 *w*/*w*). The mixed solid material is then blended with 30.37% brine in a ratio of 1:1.77 (*w*/*w*). This mixture is evenly packed into a fermentation tank and fermented at 48 °C for the first 10 days, followed by 20 days at 35 °C. On the first day of fermentation, 0.1% of papain and neutral protease is added. *T. halophilus* is cultivated in MRS broth containing 5% sodium chloride and cultured at 30 °C for 48 h. On the 10th day of fermentation, the *T. halophilus* is inoculated into the soy fish paste (10^7^ CFU/g). The preparation of soy fish skin paste is similar to that of soy fish paste, except that minced fish meat is replaced with minced grass carp skin. Samples of the soy fish paste and soy fish skin paste are collected regularly for further analysis.

### 2.3. Determination of pH, Total Acidity and Amino Nitrogen

The pH of samples was measured using a pH meter (FE28-Standard, Mettler Toledo, Shanghai, China). Total acidity and amino nitrogen were determined using the formaldehyde titration method, in accordance with the method described by Liu et al. [9],with slight modifications. A 20 mL sample of diluted soy fish paste or soy fish skin paste was mixed with 60 mL of distilled water. The mixture was titrated with 0.05 mol/L NaOH to pH 8.2, and the volume of NaOH consumed was recorded to calculate total acidity. Then, 10 mL of formaldehyde solution was added, and the mixture was titrated with 0.05 mol/L NaOH to pH 9.2. The volume of 0.05 mol/L NaOH consumed in this second titration was used to calculate the amino nitrogen content.

### 2.4. Determination of Total Volatile Basic Nitrogen (TVB-N)

TVB-N was determined using a micro-diffusion method [10], with slight modifications. A 1 g paste sample was mixed with 10 mL of deionized water. After centrifugation, 1 mL of the supernatant was added to the outer chamber of a diffusion dish. In the center of the diffusion dish, 1 mL of 20 g/L boric acid solution and one drop of methyl red–bromocresol green indicator were added. Next, 1 mL of saturated potassium carbonate solution was quickly added to the notch of the frosted glass lid, which was then rotated slightly. The dish was placed in a 37 °C incubator for 2 h. Then, the lid was taken off. The solution was titrated with 0.01 mol/L hydrochloric acid standard solution.

### 2.5. Determination of Thiobarbituric Acid Reactive Substance (TBARS) Values

The TBARS value was determined using the method of Xu et al. [11], with slight modifications. A 1 g paste sample was mixed with 5 mL of thiobarbituric acid solution, then boiled in a water bath for 20 min and cooled to room temperature. The mixture was centrifuged, and the absorbance of the supernatant was measured at 532 nm and 600 nm. The TBARS value was calculated using the following formula:

$$TBARS\left(mg/100g\right)=\left(A532-A600\right)/155\times 72.06\times 100∕10$$

### 2.6. Determination of Biogenic Amines (BAs)

The determination method for BAs uses a dansyl chloride derivatization followed by high-performance liquid chromatography (HPLC) analysis, as described by Guo et al. [12]. For sample preparation, 1 mL of the sample was mixed with 200 μL of sodium hydroxide solution (2 mol/L), 300 μL of saturated sodium bicarbonate solution, and 2 mL of dansyl chloride solution (10 mg/mL) dissolved in acetonitrile. The mixture was incubated at 45 °C in the dark for 45 min, then cooled to room temperature. Subsequently, 100 μL of ammonia solution (25%) was added, and the mixture was left in the dark for 30 min. After derivatization, the solution was diluted to 5 mL with acetonitrile, centrifuged at 3000 r/min for 10 min, and the supernatant was filtered through a 0.22 μm membrane for HPLC analysis.

HPLC analysis was performed using an LC-16 system (Shimadzu Corporation, Kyoto, Japan) equipped with an Agilent ZORBAX Eclipse Plus C18 column (4.6 × 250 mm, 5 µm, Agilent Technologies, Inc., Beijing, China). The mobile phase consisted of water (A) and acetonitrile (B), with the initial proportion of B set at 60%. This was increased to 75% within 15 min, 85% within 22 min, and 90% within 25 min. The proportion was held at 90% for 3 min before gradually returning to 60%. The detection wavelength is set at 254 nm, with a UV detector used for detection.

### 2.7. Electronic Tongue Analysis

For electronic tongue analysis, soy fish paste or soy fish skin paste is diluted and placed in a chromatography cabinet at 4 °C to cool for 12 h. After cooling, the top layer of oil and bottom layer of solids are removed, and the middle liquid layer is filtered. This filtered liquid is then analyzed using a taste analysis system (TS-5000Z, Insent, Atsugi, Japan). Specific parameters and methods can be referenced from the method by Wu et al. [13].

### 2.8. Electronic Nose Analysis

The electronic nose experiment is based on the method of Wang et al. [14], with slight modifications. The flavor characteristics of the samples were analyzed using an electronic nose (PEN33, Airsense Analytics Inc., Schwerin, Germany). This instrument consists of a 10-sensor array, a gas filtration device, and a data acquisition system, with each sensor having different sensitivities to volatile compounds. Soy fish paste or soy fish skin paste is placed in a 20 mL headspace vial. The mixture should be vigorously shaken before extraction to ensure uniformity during the process. Prior to use, the probe is cleaned with filtered air, and the electronic nose’s inlet is inserted into the sample vial. The measurement time is 100 s. After each measurement, the probe is cleaned for 20 s before proceeding to the next sample.

### 2.9. GC-IMS Analysis

The flavor compounds in soy fish paste and soy fish skin paste were analyzed using headspace–gas chromatography–ion mobility spectrometry (GC-IMS) (FlavourSpec, Hanon Advanced Technology Group Co., Ltd., Jinan, China) [15]. A 2 g sample was placed in a 20 mL headspace vial and incubated at 60 °C for 10 min, after which 1000 µL of headspace was automatically injected using a heated syringe at 85 °C. Gas chromatography was performed using an MXT-WAX column (30 m, 0.53 mm ID, 1 µ). The carrier gas was set to a flow rate of 2 mL/min. Analytes were eluted and separated at 45 °C, then ionized in the IMS ionization chamber with a 3H ionization source in positive ion mode. The 9.8 cm drift tube was operated at a constant voltage of 5 kV and a helium flow rate of 150 mL/min at 45 °C.

### 2.10. Statistical Analysis

In the experiments above, three biological replicates were used for analysis. Data analysis was conducted using Microsoft Excel 2021, SPSS Statistics 22, and Origin 2021 for statistical processing and visualization.

## 3. Results and Discussion

### 3.1. Physicochemical Properties of Fermented Soy Fish Paste and Soy Fish Skin Paste

**Figure 1:**
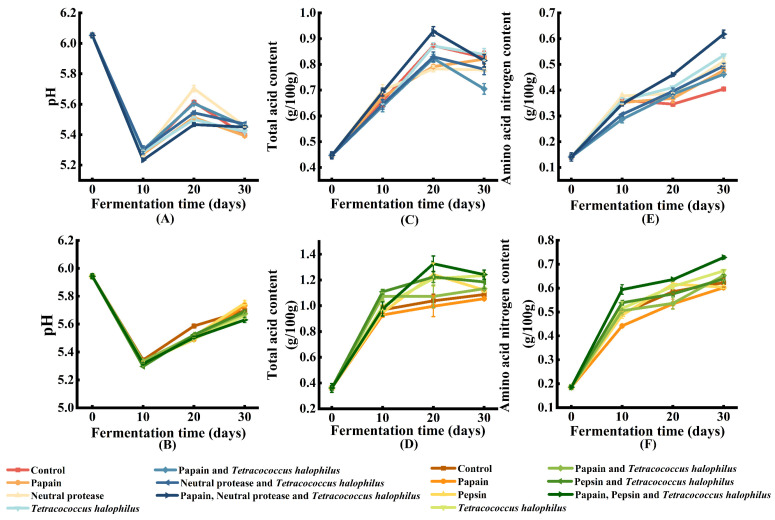
Physicochemical indexes of soy fish paste and soy fish skin paste (**A**) pH change in soy fish paste; (**B**) pH change in soy fish skin paste; (**C**) total acid change in soy fish paste; (**D**) total acid change in soy fish skin paste; (**E**) amino acid nitrogen change in soy fish paste; (**F**) amino acid nitrogen change in soy fish skin paste.

The pH, total acidity, and amino nitrogen content are key indicators of the quality of fermented condiments. Variations in these properties reflect changes in sauce quality over time. Figure 1 presents the physicochemical properties of soy fish paste and soy fish skin paste. As shown in Figure 1A,B, the pH levels of both pastes decreased during the first 10 days of fermentation, followed by a slight increase between days 10 and 20. By day 30, minor differences in pH were observed among the groups. The initial decline in pH was likely due to the activity of amylase in *Aspergillus oryzae*, which hydrolyzed the starch in the soybeans. This process provided monosaccharides and disaccharides that were further utilized by microbes, leading to a drop in pH. The subsequent rise in pH corresponds to changes in total acidity, as shown in Figure 1C,D. Total acidity increased initially and then decreased, mirroring the pH trend. By the end of fermentation, the pH of the fish by-product paste remained within the range of 5 to 6, which is comparable to that of soy sauce and generally aligns with consumer taste preferences [16].

Amino acid nitrogen (ANN) is a key indicator in fermented condiments, reflecting the degree of protein hydrolysis and contributing to the umami flavor of products. As shown in Figure 1E,F, the ANN levels in both the soy fish paste and soy fish skin paste continuously increased during fermentation. On day 30, the soy fish paste (Figure 1E) that had been treated with papain, neutral protease, and *T. halophilus* showed the highest ANN content, reaching 0.62 g/100 g, which was 0.22 g/100 g higher than the control group, marking a 342.86% increase from the initial level. In the soy fish skin paste (Figure 1F), the group treated with papain, pepsin, and *T. halophilus* had the highest amino nitrogen content, reaching 0.73 g/100 g, 0.11 g/100 g higher than the control group and representing a 284.21% increase from the initial level. Over the 30-day fermentation period, the ANN content in the soy fish skin paste increased more than in the soy fish paste. This difference may be attributed to the protein composition of fish meat and fish skin differs, with the former primarily composed of myofibrillar proteins and the latter mainly consisting of collagen. Mixed fermentation with enzymes and microbes has a stronger hydrolytic effect on collagen. Generally, the traditional fish sauce fermentation process shows a low concentration of ANN around 30 days, typically in the range of 0.2–0.4 g/100 mL [17]. However, in our study, unlike traditional fish sauce, koji was added. The *A. oryzae* in koji can produce proteases, which accelerated the fermentation process. Therefore, within the same fermentation period, the ANN content in the soy fish paste and soy fish skin paste in this study was higher. In addition, in the second stage of fermentation, inoculation with *T. halophilus* and the addition of proteases can further enhance the release of ANN, thereby shortening the fermentation period.

### 3.2. Safety Evaluation of Soy Fish Paste and Soy Fish Skin Paste

Soybeans and fish are rich in lipids, proteins, and other nutrients. During fermentation, these compounds break down and not only contribute flavor compounds such as umami and aroma to the sauce but may also produce compounds that are potentially harmful to health, such as volatile basic nitrogen (TVB-N) and biogenic amines (BAs). Whether the enzymes and microorganisms that promote the breakdown of substrates pose food safety risks to the sauce is an issue that needs consideration.

#### 3.2.1. Total Volatile Basic Nitrogen (TVB-N) in Soy Fish Paste and Soy Fish Skin Paste

**Figure 2:**
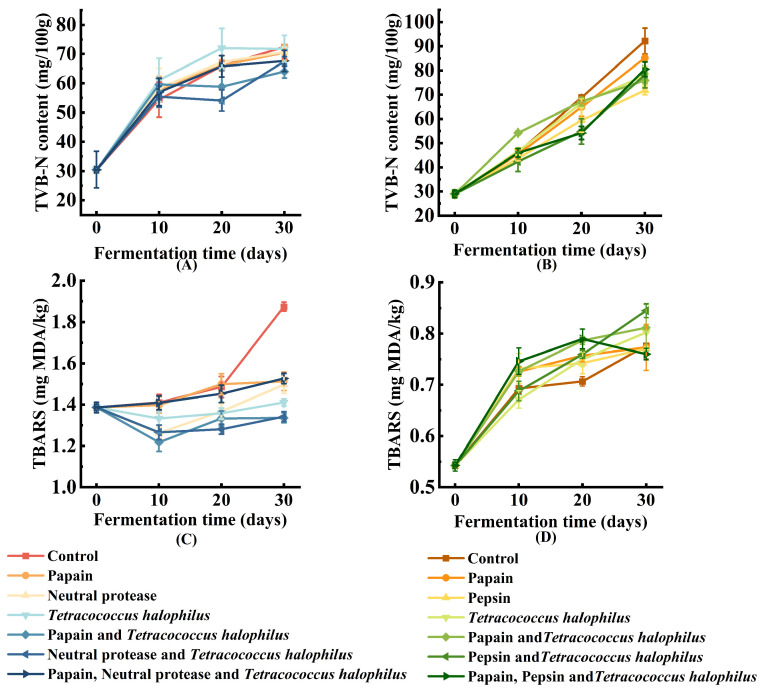
Safety evaluation of soy fish paste and soy fish skin paste (**A**) total volatile base nitrogen (TVB-N) of soy fish paste; (**B**) total volatile base nitrogen (TVB-N) of soy fish skin paste; (**C**) changes in TBARS values of soy fish paste; (**D**) changes in TBARS values of soy fish skin paste.

The measurement of TVB-N is a common and effective method for analyzing the spoilage degree of protein-rich food. These compounds are toxic and cause considerable flavor changes. As shown in Figure 2, TVB-N levels increase during the fermentation process. On the 30th day of fermentation, the fermented fish paste group (Figure 2A) with added papain and *T. halophilus* had the lowest TVB-N value, while the naturally fermented group had the highest. Recommendations for TVB-N limits vary depending on food freshness standards. For fresh meat, fish and similar raw materials, TVB-N levels are typically required to remain below 40 mg/100 g [18]. Fermentation, however, causes a significant increase in TVB-N content, with levels in fish often reaching up to 100 mg/100 g [19].

The TVB-N content varies in pastes fermented using different fish by-products. After 30 days of fermentation, the TVB-N content of soy fish paste ranges between 60 and 75 mg/100 g, while soy fish skin paste exhibits higher levels, ranging from 70 to 95 mg/100 g During this period, the TVB-N levels increased by 137.60% in the naturally fermented fish paste group, while the naturally fermented fish skin paste group exhibited a rise of 218.16%, likely due to differences in protein content between the two raw materials. The more extensive degradation of fish skin during fermentation compared to fish meat may explain the higher TVB-N levels in fish skin paste.

The accumulation of TVB-N is strongly influenced by enzyme activity and microbial composition [20,21]. Selecting appropriate enzymes is, therefore, essential for maintaining low TVB-N levels in fermented pastes. In enzyme–bacteria co-fermented groups, TVB-N levels were lower compared to those fermented with *T. halophilus* alone or with a single protease. In fermented fish skin paste (Figure 2B), the addition of pepsin yielded the lowest TVB-N levels, while the naturally fermented group exhibited the highest. This suggests that enzyme–bacteria co-fermentation could suppress spoilage bacteria, as evidenced by the reduced TVB-N levels in the co-cultured groups.

#### 3.2.2. Lipid Oxidation in Soy Fish Paste and Soy Fish Skin Paste

The TBARS value is a widely used indicator of lipid oxidation in fish products, as elevated oxidation levels can significantly affect the quality of fish paste. As shown in Figure 2C, the final TBARS values in all groups of fermented soy fish paste, except for the naturally fermented group, were below 1.5 mg MDA/kg at the end of the 30-day fermentation period. In contrast, the TBARS values in soy fish skin paste increased greatly between days 0 and 10 (Figure 2D), then stabilized for the remainder of the fermentation. By day 30, the TBARS values in all soy fish skin paste samples were below 1 mg MDA/kg. Overall, the TBARS values of soy fish paste and soy fish skin paste samples were generally below 2 mg MDA/kg, which is considered low compared to the levels reported in existing publications on fermented fish products [22]. This indicates a relatively low level of lipid oxidation in the two fermented pastes.

#### 3.2.3. BAs in Soy Fish Paste and Soy Fish Skin Paste

**Figure 3:**
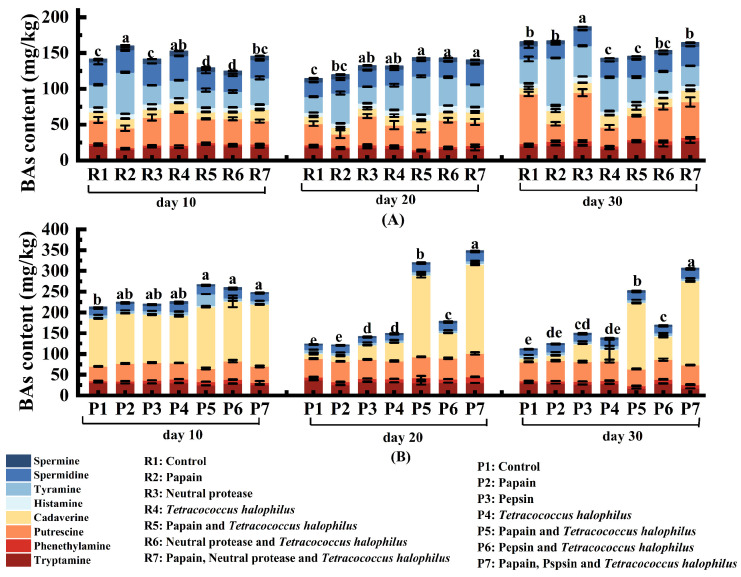
Analysis of the biogenic amine content of soy fish paste and soy fish skin paste (**A**) soy fish paste ferments biological amine; (**B**) soy fish skin paste ferments biological amine. Noted: the lowercase letters (a, b, c, etc.) in the figure indicate the significance of differences in total BAs between the control group and the groups with added enzymes or starter cultures at the same fermentation time point.

The changes in BAs in soy fish paste and soy fish skin paste are presented in Figure 3. By day 30, the total BAs in naturally fermented soy fish paste reached 165.82 mg/kg (Figure 3A), compared to 112.18 mg/kg in naturally fermented soy fish skin paste (Figure 3B). The highest total BAs content in soy fish paste, recorded at 186.68 mg/kg, occurred in the group treated with neutral protease.

In contrast, soy fish skin paste showed its highest BA level, 306.57 mg/kg, in the samples treated with papain, pepsin, and *T. halophilus*. The elevated BA level in soy fish skin paste can be attributed to the increased cadaverine content. The combination of papain and *T. halophilus* likely enhanced protein hydrolysis, resulting in higher cadaverine production. Despite this rise, cadaverine levels in soy fish skin paste remained significantly below the reported IC_50_ of approximately 40 mM, as identified in a cytotoxicity study by Rio et al. [23]. In addition, commercial soy sauce contains total biogenic amines ranging from 41.7 to 1357 mg/L [24], suggesting that the total amine content in both soy fish paste and soy fish skin paste falls within the acceptable range.

Histamine and tyramine, both toxic BAs, were detected at varying levels in the samples. In soy fish paste, histamine levels ranged from 5.86 to 7.97 mg/kg, while soy fish skin paste contained 1.16 to 2.07 mg/kg. Tyramine levels in soy fish paste vary between 27.28 and 66.49 mg/kg compared to 5.39 to 7.48 mg/kg in soy fish skin paste. For comparison, commercial fish sauce contains significantly higher concentrations, with histamine ranging from 100 to 1500 mg/kg [25] and tyramine from 80.14 to 234.60 mg/kg [26]. These results show that histamine and tyramine levels in soy fish paste and soy fish skin paste are lower than those typically found in fish sauce and soy sauce. The use of *T. halophilus*, known for its ability to degrade biogenic amines and inhibit spoilage bacteria, further reduce tyramine levels. In soy fish paste inoculated with *T. halophilus*, tyramine content was lower than in the naturally fermented samples. Similarly, in soy fish skin paste, tyramine levels in the *T. halophilus*-treated group were 0.51 mg/kg lower than in the naturally fermented group. The combination of papain and *T. halophilus* resulted in tyramine levels 0.38 mg/kg lower than those observed in the papain-only group.

### 3.3. Taste and Odor Analysis of Soy Fish Paste and Soy Fish Skin Paste

#### 3.3.1. Electronic Tongue Analysis

**Figure 4:**
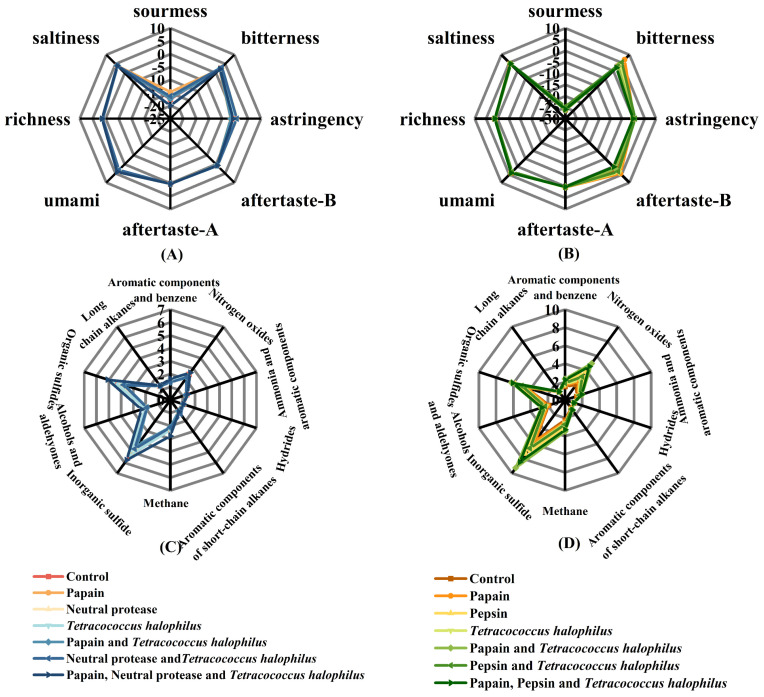
Taste and odor of soy fish paste and soy fish skin paste (**A**) E-tongue radar map of soy fish paste; (**B**) E-tongue radar map of soy fish skin paste; (**C**) E-nose radar map of soy fish paste; (**D**) E-nose radar map of soy fish skin paste.

As condiments, the sensory qualities of the soy fish paste and soy fish skin paste in this study also need attention. To analyze the non-volatile and volatile compounds in the samples, we employed E-tongue and E-nose devices, respectively. The electronic tongue analysis for soy fish paste and soy fish skin paste is presented in Figure 4. The sourness detected in soy fish paste (Figure 4A) is linked to organic acids formed during primary fermentation. Research indicates that sweetness can mitigate perceived sourness intensity [27], and the breakdown of fish muscle proteins into sweet-tasting amino acids plays a role. Differences in amino acid hydrolysis among groups probably explain the variations in sourness intensity across soy fish paste samples. Bitterness, a key focus for protein-rich fermentation products, arises when proteases hydrolyze proteins into polypeptides containing hydrophobic amino acids at the terminals. For instance, papain-generated polypeptides with hydrophobic C-terminus groups increase bitterness in the product [28]. Conversely, research by Idowu and Benjakul demonstrates that pepsin can cleave hydrophobic peptides at the C-terminus under specific conditions [29]. Accordingly, the bitterness level in soy fish skin paste (Figure 4B) is notably higher in groups with papain alone, while in soy fish paste samples with pepsin, bitterness is comparatively lower.

#### 3.3.2. Electronic Nose Analysis

The radar charts for the electronic nose of soy fish paste and soy fish skin paste are shown in Figure 4C,D. The primary flavor characteristic of soy sauce is its savory aroma, derived from volatile compounds such as alcohols, aldehydes, ketones, nitrous oxide, and aromatic hydrocarbons [30]. Among these, the electronic nose sensors are especially sensitive to alcohols, aldehydes, and aromatic compounds like benzene. The sensor readings indicate higher levels of alcohols and aldehydes in the soy fish skin paste supplemented with papain and *T. halophilus*. Xu et al. [31] reported that papain addition significantly raises the aldehyde content. The combined presence of *T. halophilus* and papain likely enhances this effect. These findings suggest that incorporating protease can help maintain the desired flavor profile, even when the fermentation time is reduced.

The soy fish paste and soy fish skin paste show higher sensor responses for nitrogen oxide, inorganic sulfide, and organic sulfide. Among these, inorganic sulfide is a recognized indicator of spoilage [32]. Notably, soy fish skin paste contains more inorganic sulfide than soy fish paste, which aligns with the observed differences in their TVB-N contents. The characteristic fishy odor in these products arises from sulfur compounds and nitrogen oxides, with lipid oxidation playing a contributory role [33]. The soy fish skin paste exhibits higher nitrogen oxide levels than soy fish paste, in line with previously noted lipid oxidation rates. Additionally, compounds generated through protease hydrolysis can further intensify the fishy aroma. In soy fish skin paste, nitrogen oxide content is lower in the pepsin and *T. halophilus* group compared to the papain and *T. halophilus* group, likely due to pepsin’s action on hydrophobic residues. The fishy odor may stem from short peptides with internal hydrophobic groups formed through protein hydrolysis. Consequently, soy fish skin paste with added pepsin and *T. halophilus* has reduced nitrogen oxide content.

While the electronic tongue and nose provide valuable insights into the non-volatile and volatile compounds in these products, they also have limitations. These instruments offer generalized responses to compound categories rather than specific compound identification, which can make detailed interpretation challenging. Additionally, they cannot fully capture the complexity of human sensory perception. Therefore, future evaluations of such products should involve trained sensory analysts to complement instrumental analyses and provide a more comprehensive assessment of their sensory qualities.

#### 3.3.3. Fingerprint Analysis of VOCs

**Figure 5:**
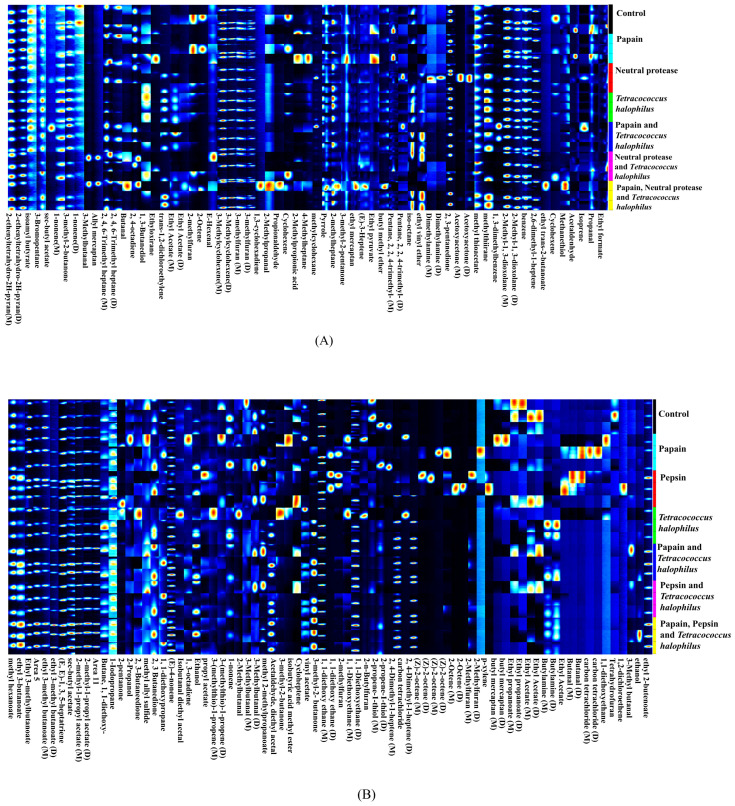
A comprehensive study for odor compounds of soy fish paste and soy fish skin paste (**A**) fingerprints of VOCs in different soy fish paste; (**B**) fingerprints of VOCs in different soy fish skin paste.

Figure 5 illustrates the volatile compound profiles of soy fish paste and soy fish skin paste. In soy fish paste (Figure 5A), 50 compounds were identified, including esters (7), ketones (5), alkanes (7), aldehydes (7), alcohols (4), alkenes (10), and 10 other compounds. In soy fish skin paste (Figure 5B), 56 compounds were detected, comprising esters (11), ketones (3), alkanes (8), aldehydes (5), alcohols (6), alkenes (14), and 9 other compounds. Both products are rich in esters, aldehydes, and alkenes, with aldehydes and esters serving as the primary flavor compounds.

Aldehydes are important flavor compounds in food, often formed through fatty acid oxidation. In fermented soy fish skin paste, 2-methylbutanal contributes nutty, roasted, and fruity aromas and has been recognized as a key aroma compound in fermented bean pastes and soy sauce-based seasonings. According to Zhou et al. [34], high-fat content promotes the formation of 2-methylbutanal. Since both soybeans and fish skin are rich in lipids, this could explain the presence of 2-methylbutanal in soy fish skin paste.

Esters also play a critical role in enhancing the flavor of fermented foods. Ethyl acetate, a compound with a fruity aroma, is commonly found in soy sauce. In this study, ethyl acetate was detected in groups with the addition of *T. halophilus* and in some groups with added protease. This may be attributed to the proteases’ ability to hydrolyze proteins, which supports microbial growth and promotes complex metabolic activities, including esterification reactions, leading to ethyl acetate formation. Zou et al. reported that soy sauce fermented for only 28 days contains limited volatile compounds [35]. Despite their shorter fermentation time, soy fish paste and soy fish skin paste retain a robust flavor similar to that of longer-fermented seasonings like soy sauce. This study conducted a preliminary analysis of some VOCs in fermented soy paste, partially substituted with fish by-products. Since VOCs are critical indicators of fermented paste quality, reflecting both desirable flavors and potential off-flavors, further research is necessary to delve deeper into their characteristics. Future studies could incorporate advanced tools such as olfactory detection combined with professional panel evaluations to identify the key VOCs responsible for flavor profiles. Additionally, exploring the interplay between starter cultures, microbial communities in the paste, and their metabolic contributions to VOC formation during the fermentation process would provide valuable insights into optimizing product quality.

## 4. Conclusions

This study highlights the potential of partially substituting soybean protein with protein-rich freshwater fish by-products, specifically grass carp minced fish and fish skin, in fermentation for producing high-value seasonings. Through the integration of microbial fermentation with enzymatic treatment, this research study achieved enhanced safety, good flavor profiles, and improved overall quality in soy fish paste and soy fish skin paste. The results demonstrate a sustainable approach to utilizing fish by-products, offering a promising pathway for innovative and value-added condiment production. While this study provides a good foundation, further research is required to develop scalable fermentation processes and conduct comprehensive sensory evaluations to bring such paste products to industrial production.

## Data Availability

The data that support the findings of this study are available from the corresponding author upon reasonable request.
